# Perioperative intravenous fluid prescribing: a multi-centre audit

**DOI:** 10.1186/s13741-015-0025-9

**Published:** 2015-12-18

**Authors:** Benjamin Harris, Christian Schopflin, Clare Khaghani, Mark Edwards, on behalf of collaborators from the Southcoast Perioperative Audit and Research Collaboration (SPARC)

**Affiliations:** Academic Department of Critical Care, Queen Alexandra Hospital, Southwick Hill Road, Cosham, Portsmouth, PO6 3LY UK; Anaesthetic Department, Queen Alexandra Hospital, Southwick Hill Road, Cosham, Portsmouth, PO6 3LY UK; Anaesthetic Department, Royal Hampshire County Hospital, Romsey Road, Winchester, , Hampshire SO22 5DG UK; Anaesthetic Department Mail Point 24, University Hospital Southampton NHS Foundation Trust, Tremona Road, Southampton, SO16 6YD UK

**Keywords:** Intravenous fluids, Electrolytes, Perioperative, Anaesthesia, Surgery, Postoperative

## Abstract

**Background:**

Excessive or inadequate intravenous fluid given in the perioperative period can affect outcomes. A number of guidelines exist but these can conflict with the entrenched practice, evidence base and prescriber knowledge. We conducted a multi-centre audit of intraoperative and postoperative intravenous fluid therapy to investigate fluid administration practice and frequency of postoperative electrolyte disturbances.

**Methods:**

A retrospective audit was done in five hospitals of adult patients undergoing elective major abdominal, gastrointestinal tract or orthopaedic surgery. The type, volume and quantity of fluid and electrolytes administered during surgery and in 3 days postoperatively was calculated, and electrolyte disturbances were studied using clinical records.

**Results:**

Data from four hundred thirty-one patients in five hospitals covering 1157 intravenous fluid days were collected. Balanced crystalloid solutions were almost universally used in the operating theatre and were also the most common fluid administered postoperatively, followed by hypotonic dextrose-saline solutions and 0.9 % sodium chloride. For three common uncomplicated elective operations, the volume of fluid administered intraoperatively demonstrated considerable variability. Over half of the patients received no postoperative fluid on day 1, and even more were commenced on free oral fluids immediately postoperatively or on day 1. Postoperative quantities of sodium exceeded the recommended amounts for maintenance in half of the patients who continued to receive intravenous fluids. Potassium administration in those receiving intravenous fluids was almost universally inadequate. Hypokalaemia and hyponatraemia were the common findings.

**Conclusions:**

We documented the current clinical practice and confirmed that early free oral fluids and cessation of any intravenous fluids is common postoperatively in keeping with the aims of enhanced recovery after surgery programmes. Excessive sodium and water and inadequate potassium in those given intravenous fluids postoperatively is common and needs to be investigated. The variation in intraoperative fluid volume administration for three common procedures is considerable and in keeping with other international studies. Future trials of fluid therapy should include the intraoperative and postoperative phases.

## Background

The fundamental goal of any intravenous fluid therapy is to restore and maintain normal fluid and electrolyte physiology in situations where patients are unable to control their own fluid intake, while minimising the risk of fluid-related complications. Excessive or inadequate intravenous fluid therapy in surgical patients is associated with adverse outcomes and can cause significant harm (Minto and Mythen 2015; Lobo et al. 2001).

Most of the intravenous fluid that surgical patients receive during their admission is delivered in the postoperative stage and most of the responsibility for prescribing these intravenous fluids lies with junior surgical staff (Walsh and Walsh 2005). The National Confidential Enquiry into Perioperative Deaths (1999) attributed significant perioperative morbidity and mortality to errors in fluid and electrolyte administration (Callum et al. 1999). Despite this, the evidence base around fluid administration in the postoperative period is still very limited (Gonzalez-Fajardo et al. 2009).

There have been a number of guidelines and recommendations regarding perioperative intravenous fluid therapy in recent years, and these aim to provide a basis for good practice and provide a resource for quality improvement (National Institute for Health and Care Excellence 2013; Powell-Tuck et al. 2011). They highlight those areas of the hospital, such as general surgical wards, where errors are more likely than in operating theatres and patients may be at greatest risk of inappropriate and potentially harmful intravenous fluid therapy (Minto and Mythen 2015; National Institute for Health and Care Excellence 2013). Intravenous fluid guidelines have to combine physiological principles with entrenched historical and variable practice, an inadequate and often contradictory evidence base, a multitude of intravenous solutions that often vary in availability between individual hospitals and a deficiency of prescribing knowledge among many medical staff. While there is some debate about the content of these guidelines (Woodcock 2014; Soni 2009) and a recognition that deviations from guidelines may be an appropriate response to a patient’s clinical situation, for the majority of elective cases, the guideline recommendations represent an appropriate standard of care against which to audit.

It has been suggested that despite the guidelines and the evidence, intraoperative and postoperative fluid prescribing practice is both variable and suboptimal (Minto and Mythen 2015; National Institute for Health and Care Excellence 2013; Powell-Tuck et al. 2011). We conducted a multi-centre audit of intraoperative and postoperative intravenous fluid therapy in adult elective major surgery patients to investigate the following: current intraoperative intravenous fluid administration practice, current postoperative intravenous fluid administration practice (both compared to recent guidelines) and frequency of postoperative electrolyte disturbances. The data produced will be used for future quality improvement projects.

## Methods

We undertook a multi-centre retrospective observational audit across five Wessex deanery hospitals. This included four district general hospitals and one university teaching hospital. Appropriate approval was obtained via individual hospital audit departments, and the investigation was co-ordinated by one local lead investigator in each hospital. We aimed to investigate 100 cases in each hospital. Inclusion criteria were adult patients who underwent elective major surgery during the year 2013 as defined by the BUPA complexity categories. These ascend in surgical complexity order from major, major+ and complex major operation (CMO) (BUPA Schedule of Procedures 2005). Specialities included orthopaedic, upper and lower gastrointestinal, urological and gynaecological surgeries. All patients irrespective of postoperative destination were included. Exclusion criteria were non-elective surgery, minor or intermediate surgery, day case surgery and patient age less than 18 years.

A list of all patients meeting the inclusion criteria was obtained in each hospital, and the first 100 patients in chronological order (January 2013 onwards) were selected. In high-volume centres, the target of identifying 100 patients was reached within the first 4 months of the year. In lower volume centres, to identify the required 100 subjects, operations up to October 2013 were included. Once 100 patients were identified in each centre, collection of identifying details ceased and the medical notes were requested. Hospital C was a very high volume centre, and in order to improve the overall number of patients in the audit, an additional 50 patients were identified in hospital C and their notes requested.

From the medical notes, where available, patient and procedure characteristics including age, weight (actual body weight recorded on the pre-operative assessment or anaesthetic paperwork prior to the operation) and operation type were recorded. For each patient, we collected data on volume and fluid type administered, quantities of sodium, chloride and potassium administered per kilogram body weight per day, calculated fluid balance based on input/output charts (fluid prescription charts and output charts recording urine, drain and other losses) and serum electrolyte measurements. We defined hyponatraemia as a serum sodium of less than 135 mmol.l^−1^ and hypokalaemia as a serum sodium of less than 3.5 mmol.l^−1^. Data collection covered the period from the day of the operation up to and including postoperative day 7, and the day after intravenous fluids were completely ceased or the day of discharge if this came first. The ‘standards’ used for this audit were the GIFTASUP guidelines (Powell-Tuck et al. 2011) that advocate early resumption of oral intake, early cessation of intravenous fluids and careful attention to fluid balance. The volume and type of intravenous fluid together with the quantity of electrolytes administered perioperatively was compared with the recommended values (Powell-Tuck et al. 2011). These recommendations are, for the maintenance of homeostasis, approximately 1500–2500 ml water per day (interpreted by us as 25–30 ml.kg.day^−1^ of water) (Powell-Tuck et al. 2011). For sodium and potassium, requirements for maintenance should be close to the reference nutrient intake (RNI) (Powell-Tuck et al. 2011). Values of 50–100 mmol per day for sodium and potassium 40–80 mmol per day are recommended (interpreted by us as 0.8–1.2 mmol.kg.day^−1^). These quantities are very similar to other more recent intravenous fluid guidelines (National Institute for Health and Care Excellence 2013).

Each hospital was assigned a random letter (A–E) with the identity of each hospital known only to the principal investigator. No patient identifiable data was recorded and kept, and all recorded data was anonymised and encrypted using commercially available software. Data in figures is expressed either as absolute numbers with percentages or as measures of central tendency (mean or median). Measures of spread used were standard error of the mean or interquartile range, and this is indicated in the figure legends. We did not undertake any further statistical tests so as not to deviate from the observational nature of the audit.

## Results

**Fig. 1:**
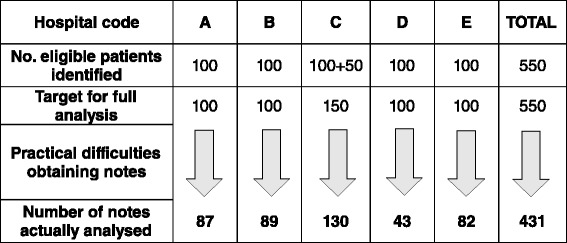
Flow chart demonstrating the target number of patients at each hospital and the number successfully included in the audit

Data were collected for 431 subjects. Although we requested the notes of the first 100 (150 in hospital C) patients in each hospital, during the analysis period, a number of patient notes that were requested were not available due to administrative reasons. These included being lost or in use at a different hospital department. Therefore, the final number of patient records analysed was 431 (Fig. 1).

The median age was 78 years (range 20–93 years) and median weight was 79 kg (range 36–210 kg). Forty-three percent of the patients underwent orthopaedic surgery, 23.5 % upper gastrointestinal and 33.5 % lower gastrointestinal, gynaecological or urological surgery. Table 1 summarises the number of cases according to speciality, category of surgical complexity and postoperative destination.

**Table 1:** The number of patients in each surgical speciality, BUPA complexity category and immediate postoperative destination clinical area

		Surgical speciality			
		Lower GI, gynaecology and urology	Orthopaedics	Upper GI	Total
BUPA category and postoperative clinical area	CMO	57	110	63	230
	ICU	12 (21 %)	3 (2.7 %)	36 (57 %)	
	HDU/ward	45 (79 %)	107 (97.2 %)	27 (43 %)	
	Major+	79	73	10	162
	ICU	28 (35 %)	2 (3 %)	4 (40 %)	
	HDU/ward	51 (65 %)	71 (97 %)	6 (60 %)	
	Major	8	3	28	39
	ICU			1 (4 %)	
	HDU/ward	8 (100 %)	3 (100 %)	27 (96 %)	
	Total	144	186	101	431

BUPA category codes are defined in the text of the “Methods” section

*Abbreviations*: *GI* gastrointestinal, *CMO* complex major operation,

### Fluid volume and electrolyte quantities

The audit set out to record fluid and electrolyte quantities for each postoperative day up to and including postoperative day 7. However, the quality of fluid prescription charts and fluid balance documentation was subjectively noted by the data analysing team to become progressively less complete or even present in the medical notes as length of stay progressed. Early on in the data collection process (after analysing approximately 20 notes in each centre), it was therefore decided to limit further data collection to 3 days postoperatively only due to the paucity of data. We did continue to collect the date when free oral fluids were started because this information was easier to find in the narrative medical notes. Overall, 1157 intravenous fluid days have been included in the analysis over postoperative days 1, 2 and 3.

### Type of intravenous fluid

**Fig. 2:**
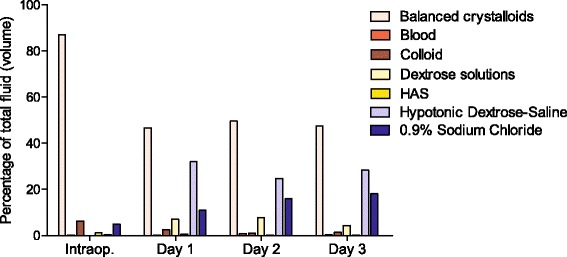
Type of intravenous fluid administered in different phases of the perioperative pathway. Balanced crystalloid solutions were the most common fluid administered at all stages of the perioperative pathway expressed as the percentage of the total volume of intravenous fluid used. Balanced crystalloids (Hartmann’s and Plasma-Lyte), hypotonic dextrose-saline solutions (0.18 and 0.45 % dextrose-saline), dextrose solutions (5 and 10 % dextrose) and HAS (human albumin solution)

During the entire time period studied, the balanced crystalloid solutions were the most common intravenous fluid (57.6 % of all fluid administered by volume). Figure 2 demonstrates that during the intraoperative period, for all types of surgery, by far the most commonly used intravenous fluids were the balanced crystalloids. In the postoperative period, balanced crystalloids were still the most common but the use of hypotonic dextrose-saline and 0.9 % sodium chloride increased markedly.

**Fig. 3:**
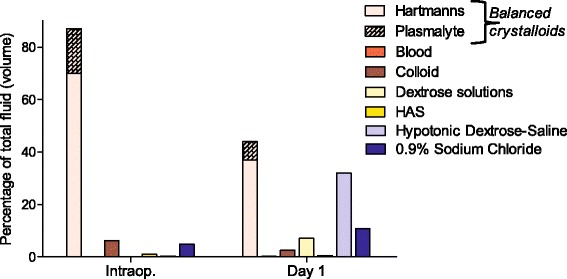
Type of intravenous fluid administered intraoperatively and on postoperative day 1. This graph illustrates the different types of balanced crystalloid that were used in this audit. It demonstrates that Hartmann’s is the dominant balanced crystalloid used in Wessex

When the balanced intravenous solutions are subdivided into Hartmann’s and Plasma-Lyte (the only two balanced solutions used in this audit), Hartmann’s was the most commonly used balanced solution intraoperatively and on postoperative day 1 (Fig. 3). This was the same for postoperative days 2 and 3 (data not shown).

### Immediate postoperative destination and type of fluid

**Fig. 4:**
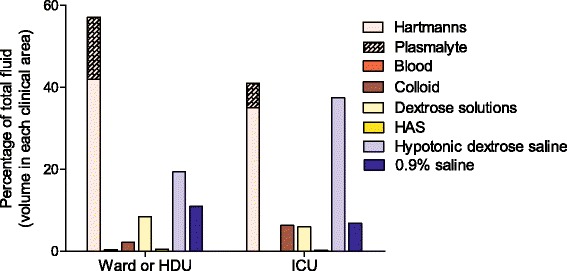
Type of fluid administered on postoperative day 1 in different clinical areas. The type of fluid administered on postoperative day 1 varied depending if the patient was cared for in a ward or ward-based HDU environment compared to an ICU environment. *HDU* high dependency unit, *ICU* intensive care unit

The type of fluid given on postoperative day 1 demonstrated a similar pattern for all clinical areas. The only difference was more hypotonic dextrose-saline was used in the intensive care unit (ICU) compared to wards or ward-based high dependency unit (HDU) (see Fig. 4).

### Volume of intraoperative fluid

**Fig. 5:**
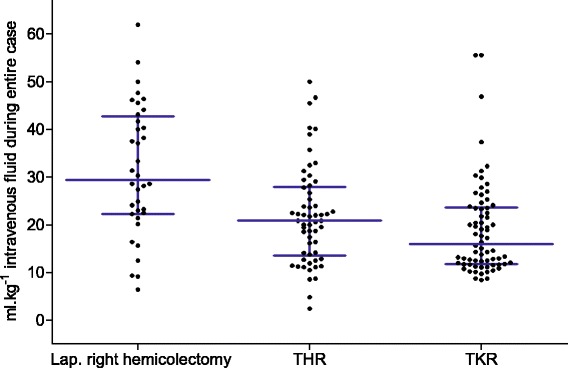
Volume of intraoperative fluid administered for three common surgeries. Volume of fluid administered expressed as ml.kg^−1^ during the entire operation. *Error bars* represent the interquartile range. The *wide horizontal line* represents the median. *Abbreviations*: *THR* total hip replacement, *TKR* total knee replacement

In keeping with the surgical severity in the group studied, almost all patients received some intravenous fluid during their time in the operating theatre. Three common procedures were laparoscopic right hemicolectomy (*n* = 36), total hip replacement (*n* = 58) and total knee replacement (*n* = 70). The volume of fluid administered intraoperatively (where recorded) for these index procedures demonstrated considerable variability (Fig. 5).

### Volume of postoperative fluid

**Fig. 6:**
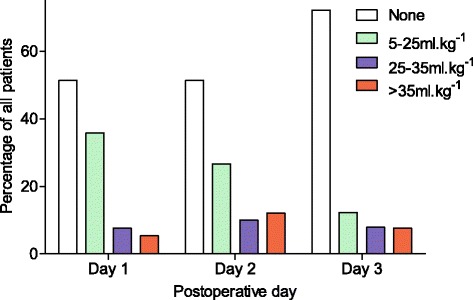
Intravenous fluid received on postoperative days 1, 2 and 3. Percentage of all patients who received different volumes of intravenous fluid expressed as ml.kg.day^−1^

**Fig. 7:**
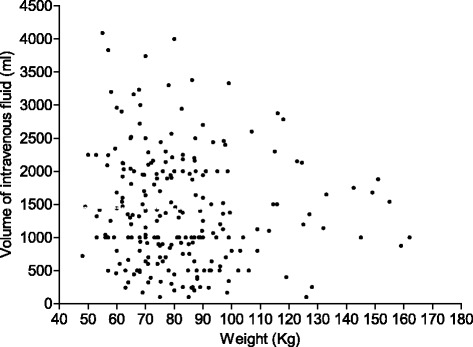
Volume of intravenous fluid given on postoperative day 1 compared to body weight. In those patients receiving intravenous fluid on postoperative day 1, there was no relationship between volume received and body weight

On postoperative days 1 and 2, over half of the patients received no intravenous fluid at all (Fig. 6). This proportion increased on day 3 to nearly three quarters. A small minority of patients received >35 ml.kg^−1^ intravenous fluid on all three postoperative days. There appeared to be no correlation between volume of fluid prescribed and body weight as shown in Fig. 7.

### Free oral fluid orders

The day when ‘free oral fluids’ were ordered by the surgical team is listed in Table 2. We recognise this as the day that intravenous fluids should be stopped as the patient is able to take oral fluids. This day was accurately recorded in the clinical notes in 67 % (*n* = 289) of all subjects. Where recorded, free fluids were ordered immediately postoperatively or on postoperative day 1 in the majority of patients (Table 2).

**Table 2:** Postoperative day that ‘free oral fluids’ were ordered by the surgical team

Perioperative day	Number of patients (%)
Immediately postoperative	185 (42.9)
Day 1	65 (15.1)
Day 2	15 (3.5)
Day 3	10 (2.3)
Day 4 or after	14 (3.2)
Not recorded in notes	142 (32.9)

### Postoperative dose of electrolytes

The quantity of sodium, potassium and chloride administered on postoperative day 1 was calculated from the known volume and type of fluid administered. Of patients who received intravenous fluid on postoperative day 1 (*n* = 248), quantities of sodium and chloride exceeded maintenance requirements in approximately half of those studied (Table 3). Almost all received less potassium than their maintenance requirements with a third of patients receiving none (Table 3). We subsequently examined patients with an apparent ongoing intravenous fluid requirement in more depth by analysing only those patients that were receiving intravenous fluids on day 2 as well as day 1 (*n* = 96). We found similar trends in day 1 sodium and potassium dosing (Table 4).

**Table 3:** Quantity of sodium, potassium and chloride given in intravenous fluids on postoperative day 1

	Quantity administered in mmol.kg^−1^			
	None (%)	0.1–0.7 (%)	*0.8–1.2* (%)	≥1.3 (%)
Sodium	7	24	*14*	55
Chloride	7	28	*17*	48
Potassium	30	68	*2*	0

For all patients receiving intravenous fluid on postoperative day 1 (*n* = 248), the percentage of those in each electrolyte dose range is listed. The dose range of 0.8–1.2 mmol.kg^−1^ is expressed in italics because this represents maintenance requirements

**Table 4:** Quantity of sodium, potassium and chloride given on postoperative day 1 in those with an ongoing need for intravenous fluids (*n* = 96)

	Quantity administered in mmol.kg−1			
	None (%)	0.1–0.7 (%)	*0.8–1.2* (%)	≥1.3 (%)
Sodium	4	14	*9*	72
Chloride	4	15	*10*	69
Potassium	25	67	*6*	0

For all patients receiving intravenous fluid on postoperative day 1, the percentage of those in each electrolyte dose range is listed. An ongoing need for intravenous fluids was defined as those receiving intravenous fluid on postoperative days 1 and 2, therefore demonstrating an ongoing need during day 1. The dose range of 0.8–1.2 mmol.kg^−1^ is expressed in italics because this represents maintenance requirements

### Electrolyte disturbances

**Fig. 8:**
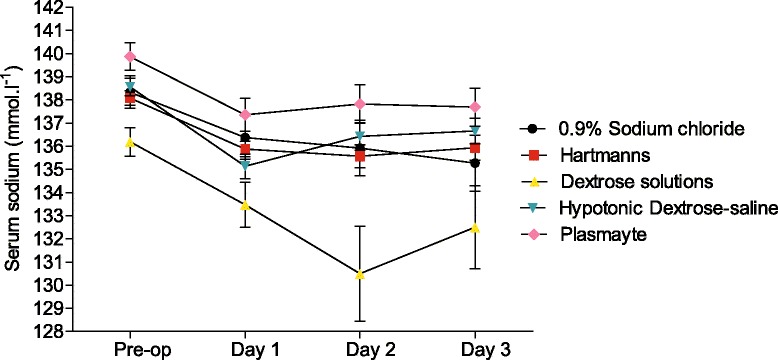
Serum sodium on each perioperative day and type of fluid given on postoperative day 1. Mean values of serum sodium on each perioperative day grouped by the type of fluid (given in the largest volume) on postoperative day 1. *Error bars* represent the standard error of the mean (SEM). Hypotonic dextrose-saline solutions (0.18 and 0.45 % dextrose-saline) and dextrose solutions (5 and 10 % dextrose)

Table 5 lists the percentage of patients with hypokalaemia in the various perioperative phases. The incidence of hyponatraemia increased after surgery (Fig. 8). It appeared most marked on postoperative day 2 in those given dextrose solutions (5 or 10 % dextrose) on postoperative day 1 compared to those given other types of fluid although the pre-operative serum sodium was lower in this group (Fig. 8).

**Table 5:** Percentage of patients with hypokalaemia in the various perioperative phases

Pre-operative value	Postoperative day 1	Postoperative day 2	Postoperative day 3
1.5 %	4 %	6 %	10 %

Percentage of patients (who had serum potassium measured) with a serum potassium less than 3.5 mmol.l^−1^ at the various perioperative stages

## Discussion

This was a large retrospective audit of current intravenous fluid prescribing practice in the five hospitals in the Wessex region. We found that early cessation of intravenous fluids in the postoperative elective surgical patient was common and in keeping with the approach promoted by enhanced recovery after surgery programmes. In those patients that continue to receive intravenous fluids, a considerable proportion appear to receive excessive or inadequate quantities of various electrolytes in relation to reference requirements. A small but important proportion appears to receive excessive volumes of water. The type of intravenous fluid administered varies according to the stage of the patient pathway. There was considerable variation in the volume of fluid administered in the operating theatre for three common elective procedures. Electrolyte disturbances were frequent and became more common as the postoperative days passed.

### Strengths of this audit

This is a large, multi-centre audit across five hospitals serving a wide geographical area with a sample population typical of most regions across the country. The included surgical procedures are common and relevant to a wide audience. The data collected has allowed a detailed analysis of the current practice, and the findings are in keeping with previous work (Minto and Mythen 2015; Callum et al. 1999; Lilot et al. 2015). The majority of recent major trials regarding intravenous fluids focus on intraoperative fluid management. However, only a small proportion of the perioperative journey is spent in the operating theatre under the direct care of an anaesthetist. Our work addresses both the intraoperative and postoperative periods.

### Limitations of this audit

#### Heterogeneity

The patients included are a very heterogeneous group, with a wide range of age, body weight, mode of anaesthesia and operation type.

### Data capture

For administrative and logistical reasons at the different institutions, we could not obtain 100 % of the notes identified leading to a potential for selection bias. Hospital C contributed relatively more patients potentially skewing the overall results.

### Data limitations

The majority of medical records in this audit were paper based and often subjectively of poor quality which may affect the results. We did not collect detailed data on individual patient perioperative risk scores in the form of ASA (American Society of Anaesthesiologists) grade or other perioperative risk scoring systems. We considered ASA grade to be insufficiently robust to determine perioperative risk, and other risk scoring systems were rarely used by anaesthetists in this audit.

When calculating intraoperative fluid administration data for the three common procedures, our ability to determine that these were uncomplicated was based on discharge summaries and anaesthetic charts. Neither of these two methods may have recorded all the technical difficulties or other ‘complications’ during the surgery that may have mandated higher than average intravenous fluid administration volumes. In addition, the length of time spent in the operating theatre was not recorded so we were unable to present intraoperative fluid administration volumes as ml.kg.h^−1^. Also, these patients were not matched in terms of perioperative risk score (e.g. ASA), co-morbidities, surgical complexity or operative time, although we did exclude non-elective patients and those with significant blood loss. Fluid balance charts were studied to help assess appropriateness of intravenous fluids. These were subjectively noted to be often of poor quality and do not record all losses, such as fluid sequestration in sepsis, so they do not provide all the information needed to assess whether the fluid administered is clinically appropriate. Fluid prescription charts were better in terms of completion.

### Study design

Volumes of fluid and quantities of sodium were calculated to be excessive in reference to the maintenance requirements in a large number of patients in the postoperative period. An unknown proportion might have been septic, bleeding or hypovolaemic requiring additional replacement over and above the maintenance requirements. A detailed notes review would be required to assess how clinically appropriate the fluid volume and sodium quantity given in each case was. Enhanced recovery pathways and fluid prescription guidelines often vary between individual hospitals and even individual surgical teams in the same institution. This may explain some of the variation in practice.

### Meaning of this audit

One of the principles of enhanced recovery after surgery protocols is an early return to oral intake to improve patient comfort, gut function and limit the detrimental effects of intravenous fluid (Guidelines for the Implementation of Enhanced Recovery Protocols 2009). Intravenous fluid guidelines (National Institute for Health and Care Excellence 2013; Powell-Tuck et al. 2011) also make reference to promoting the early return to oral intake. Over 50 % of the patients had a free oral fluid order on the day of their operation or postoperative day 1, indicating an attempt to return them to normal oral fluid intake. Of the patients, 32 % had no record of any free fluid order being made possibly because it was obvious that the patient could drink freely or that the hospitals have adopted an enhanced recovery approach and assumed free oral fluids can be given unless specifically documented otherwise. Poor medical record keeping is another possibility. At least some of these 32 % of patients would have been allowed to drink free oral fluids; therefore, the lack of documentation is unlikely to underestimate the already large number of patients with an early (day of operation or postoperative day 1) free fluid order.

The balanced crystalloid solutions were the most common postoperative fluid, with hypotonic crystalloids (dextrose-saline solutions) and 0.9 % sodium chloride as the next most common. There is increasing recognition that the traditional postoperative regime of 0.9 % sodium chloride and 5 % dextrose risks sodium, chloride and salt overload (De Silva et al. 2010). Sodium chloride (0.9 %), even when not given in excessive quantities, is associated with a variety of detrimental effects such as hyperchloraemic acidosis, reduced renal blood flow, increased chance of renal failure and increased in-hospital mortality after major abdominal surgery (Lobo 2012). Data from Wessex in 2007 found that over 70 % of postoperative fluid prescriptions were 0.9 % sodium chloride or 5 % dextrose (De Silva et al. 2010). This figure was reduced to 40 % in 2009 after a targeted education intervention (De Silva et al. 2010). It appears that this downward trend has continued as evidenced by our work.

Differences in intraoperative and postoperative fluid types is partially explained by the prescriber (anaesthetists in theatre vs junior doctors or non-anaesthetists postoperatively) and the different physiology occurring at the different time points.

Even the use of the balanced crystalloids can result in sodium overload and some hospitals and the NICE (National Institute for Health and Care Excellence 2013) advocate hypotonic dextrose-saline solutions as a means of giving water with minimal sodium to meet the maintenance requirements. It is not surprising that the greater use of hypotonic dextrose-saline solutions was seen in patients that went to the ICU postoperatively compared to HDU or ward, perhaps indicating better intravenous fluid prescribing practice or awareness of guidelines.

The inadequate doses of intravenous potassium required to meet the maintenance seen in our audit are in keeping with previous work (Lu et al. 2013) and our anecdotal observations. Although it is possible that some patients received potassium supplementation by other means (i.e. orally), the increasing incidence of hypokalaemia as the postoperative period progresses suggests that inadequate intravenous (and other) potassium supplementation is a true finding and is a problem that requires addressing. There are a number of potential reasons behind this but fear of intravenous potassium and the belief that the balanced crystalloid solutions contain adequate potassium for all patients are possible explanations. Hypokalaemia in the postoperative patient has been associated with slower return of gut function as well as other complications, and there is a suggestion that preventing hypokalaemia in the postoperative stage may improve outcomes (Lu et al. 2013).

Postoperative hyponatraemia was common and is multifactorial. It is not possible to determine the causes without a detailed notes analysis of the affected patients. As Fig. 8 demonstrates, mean serum sodium declined by 2.6 mmol.l^−1^ in the 356 subjects in whom pre- and postoperative day 1 serum sodium values were available. This is despite the vast majority of intraoperative intravenous fluid therapy consisting of balanced crystalloid solutions. Therefore, the decline in mean serum sodium compared to pre-operative values is most likely to represent the physiological stress response to surgery. This is a spectrum of changes that occur throughout various body systems (neuroendocrine, metabolic, immunological and haematological) in response to surgical incision and trauma. Neuroendocrine changes are particularly relevant to perioperative fluids because the release of catecholamines and cortisol, vasopressin and aldosterone result in retention of sodium and water (often water in excess of sodium hence hyponatraemia), loss of potassium, reduced creatinine clearance and urine output. These effects can last well beyond the operative period into the postoperative phase.

Hyponatraemia can cause a variety of neurological and other symptoms but, more importantly, has been associated with increased risk of in-hospital and long-term mortality in a variety of patient groups (Lu et al. 2013). The mean drop in serum sodium from postoperative day 1 to postoperative day 2 was 0.21 mmol.l^−1^. When only looking at those patients who received any dextrose solutions on postoperative day 1, the mean drop was 2.9 mmol.kg^−1^. This raises the possibility that the use of dextrose solutions on postoperative day 1 is associated with subsequent hyponatraemia (compared to other fluids) and should be avoided. It should be noted that the pre-operative mean serum sodium was lower in this group. With the relatively small number of subjects, the retrospective nature of the audit and the large number of contributory factors, it is not possible to suggest causation. From our limited data, it would appear that the use of hypotonic dextrose-saline on postoperative day 1 was not associated with hyponatraemia and therefore, the recommendation made in several fluid guidelines (National Institute for Health and Care Excellence 2013; Woodcock 2014) that this is the preferred fluid is reasonable based on the results of this audit.

The range of intraoperative fluid doses for three index procedures was wide and in keeping with a recent two-centre observational study in America of fluid administration for uncomplicated elective abdominal surgery with minimal blood loss (Lilot et al. 2015). This found wide variability of crystalloid administration both within and between anaesthesia providers (Lilot et al. 2015). In that study, for most procedures, 50 % of patients received 4–10 ml.kg^−1^.h^−1^ of crystalloid (corrected for urine output and estimated blood loss) but 50 % fell outside of this wide range with some patients receiving as much as 35 ml.kg^−1^.h^−1^ (Lilot et al. 2015). Although a patient’s fluid requirements will vary and depend on a number of factors, it would be surprising to see such a wide range of physiological needs during similar surgical episodes, raising the possibility that variation is due to variation in individual anaesthetists’ fluid approach.

The lack of correlation between the volume of postoperative day 1 fluids and body weight is interesting. In terms of absolute volumes given, if clinicians were calculating fluid doses on a millilitre per kilogram basis, we might expect absolute volumes to increase with body weight. We saw the opposite of this with smaller patients getting larger volumes of fluids. This suggests that practitioners are thinking in terms of ‘number of bags’ for a ‘standard’ patient and not taking into account weight.

### Unanswered questions and future research

Most intravenous fluid trials take place in the operating theatre. We have demonstrated the type of intravenous fluid prescribed varies depending on whether the patient is in the postoperative ward or in theatre. The type of prescriber is likely to be different as well. Some previous intravenous fluid research has excluded the first postoperative day from any analysis because fluid prescriptions during this period may have been complicated by surgery-induced fluid and electrolyte shifts and any postoperative fluid prescriptions completed by the anaesthetist (Walsh and Walsh 2005). Being a continuum, any future research should cover the intraoperative and postoperative phases, something that has been recognised as important by an international trial looking at fluid therapy during and after major abdominal surgery Myles PS & Wallace SK (2015). Interestingly, prostatectomies in the American study (Lilot et al. 2015) had a much narrower range of fluid administered because a fluid protocol exists. A similar fluid protocol intervention may be needed in Wessex.

Education of junior doctors can make an impact on their prescribing choices (De Silva et al. 2010) but there is always a risk that any relatively short lived intervention only lasts a limited period of time before traditional practice starts to re-establish itself. The variation in intraoperative fluid volumes observed in theatre means that there is a quality improvement work that might be required in this environment as well.

## Conclusions

Our aims were to accurately document the current clinical practice of perioperative intravenous fluid prescribing and identify key areas for improvement. There is some evidence of good practice in terms of early cessation of intravenous fluid, reduced use of 0.9 % sodium chloride compared with previous audits and early free fluid orders. However, there is clearly a need for effective implementation of intravenous fluid therapy guidelines to assist prescribers in the postoperative period prescribe appropriate quantities of sodium, potassium and water relevant to the clinical need. The wide variation of intraoperative volume for three common elective procedures is of concern and needs to be investigated further. We have identified that the suboptimal use of intravenous fluid therapy is relatively common and a potential cause of excessive complications. It is of concern that a basic principle such as appropriate potassium supplementation appears to be inadequate. The publication of prominent guidelines for all involved in fluid prescribing can only help raise basic standards, while acknowledging that fluid physiology is a complex and evolving area not fully understood even by those with expertise in this field.

## Abbreviations

IV: intravenous
kg: kilogram
l: litre
mmol.l^−1^: millimoles per litre
